# Surgical Treatment of Calcaneal Fractures by Minimally Invasive Technique Using a 2-Point Distractor Versus ORIF and Conservative Therapy—A Retrospective Multicenter Study

**DOI:** 10.3390/jcm14062015

**Published:** 2025-03-16

**Authors:** Arastoo Nia, Stefan Hajdu, Kambiz Sarahrudi, Harald Kurt Widhalm, Domenik Popp, Lukas Schmoelz, Kevin Doering, Thomas Tiefenboeck

**Affiliations:** 1 Department of Traumatology and Orthopedics, Division of Trauma Surgery, Medical University of Vienna, 1090 Vienna, Austria; 2 Department Medicine, Danube Private University, 3500 Krems an der Donau, Austria; 3 Department of Traumatology and Orthopedics, Division of Trauma Surgery, Medical University of Wiener Neustadt, 2700 Wiener Neustadt, Austria; 4 Department of Traumatology and Orthopedics, Division of Orthopedic Surgery, Medical University of Vienna, 1090 Vienna, Austria

**Keywords:** calcaneal fracture, Böhler’s angle, minimally invasive, conservative, ORIF

## Abstract

**Background:** The management of intra-articular displaced calcaneal fractures remains controversial. Both minimally invasive technique (MIT) and open reduction internal fixation (ORIF) are commonly employed, but there is no clear consensus on the optimal approach. Open surgical techniques carry a higher risk of wound complications, whereas percutaneous methods may offer less precise fracture reduction, while conservative therapy (CT) shows higher functional complications. The aim of this retrospective study was to compare the radiological and functional outcomes of three treatment methods for calcaneal fractures. **Methods:** Patients admitted to two level-one trauma centers between 2013 and 2023 were analyzed. Clinical and radiological parameters and scores were collected. The outcome measures were assessed using Böhler’s and Gissane’s angles. The clinical results were compared using the Visual Analogue Scale (VAS), the American Orthopaedic Foot and Ankle Society Hindfoot Score (AOFAS), the Maryland Foot Score (MFS), and the Parker Mobility Score (PMS) at the 12-month follow-up. **Results:** A total of 236 patients with 256 calcaneal fractures were analyzed. Among them, 175 men and 61 women were included. The mean patient age was 46.3 (min-max) years. In total, 130 patients were treated operatively (minimally invasive technique or ORIF), and 106 were treated conservatively. Böhler’s and Gissane’s angles at follow-up were significantly higher within the operated group. Complications occurred more frequently in the surgical treatment group than in the conservative treatment group, especially deep infections within ORIF (*p* < 0.001). MFS and VAS yielded better results for surgical treatment. **Conclusions:** Based on the results of this study, surgical treatment is preferable to conservative treatment due to better anatomical reduction and a slightly better functional outcome. One-year results for all three methods were comparable, showing good clinical and functional results with the surgical groups.

## 1. Introduction

Displaced intra-articular calcaneal fractures (DIACF) account for approximately 75% of all calcaneal fractures. The primary causes include falls from a height and high-energy trauma, such as road traffic accidents [1]. In early therapeutic trials, Böhler demonstrated significant results with manual reduction by plantar flexion of the foot using a wooden wedge, while Tantavisut et al. achieved success with percutaneous reduction through screw insertion as early as the 1930s [2]. DIACF constitutes the majority of calcaneal fractures. In recent decades, most surgeons have preferred open reduction and internal fixation (ORIF) for reducing and stabilizing these fractures [3,4].

However, postoperative complications, such as prolonged healing, fracture malreduction, and wound healing disorders, occur in up to 29% of cases and can lead to poor outcomes and severe disability [5,6]. Minimally invasive therapies (MIT), on the other hand, are associated with lower complication rates, although inadequate reduction of intra-articular fractures remains a limiting factor [7]. The optimal treatment strategy of displaced calcaneal fractures has been debated for decades and still remains controversial. While some studies suggest improved outcomes with surgical intervention compared to conservative therapy (CT), others fail to demonstrate a significant advantage [8,9]. Long-term studies showed that surgical treatment is not better in managing displaced intra-articular calcaneal fractures at the one-year follow-up but demonstrated certain advantages after many years [10]. Due to inconsistencies in data and recommendations, our study sought to evaluate first the advantages and disadvantages of different treatment modalities by comparing radiological and functional outcomes. Second, we sought to provide evidence-based treatment recommendations for surgeons by assessing the quality of anatomical reduction, complication rates, and functional outcomes in operatively and non-operatively treated displaced intra-articular calcaneal fractures. Third, if minimally invasive surgery does have a lower complication rate than ORIF.

## 2. Materials and Methods

This retrospective multicenter study included all patients admitted to one of two level one trauma centers with a calcaneal fracture between January 2013 and December 2023, Department of Traumatology and Orthopedics, Division of Trauma Surgery, Medical University of Vienna (Center 1) and Department of Traumatology and Orthopedics, Division of Trauma Surgery, Medical University of Wiener Neustadt (Center 2).

All patients were followed up for at least 1 year. Postoperative radiographs were obtained at 1-year follow-up. Clinical Data were obtained from the medical records included basic demographics (age, sex, Sanders type, waiting time for surgery, operation time, and complications).

### 2.1. Inclusion and Exclusion Criteria

**Figure 1:**
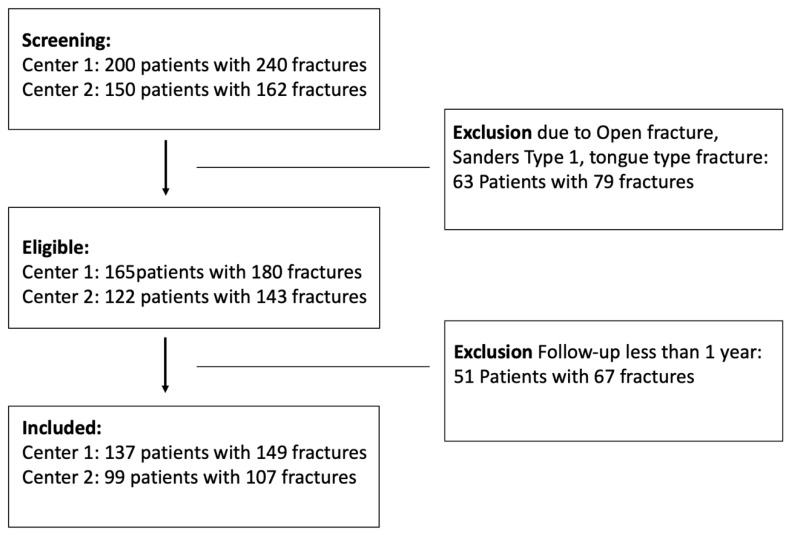
Flowchart showing the process of enrolling patients into the study.

The inclusion criteria were the diagnosis of a closed displaced intra-articular calcaneal fracture with two or more millimeters of displacement (Sanders type II–IV), treated either with ORIF or MIT or CT. All patients considered in this study had to be above 18 years of age. Specific patient exclusion criteria included a history of severe neurological deficits, previous foot surgery or trauma, primary arthrodesis or amputation, and Gustilo grade III open fractures. Patients were excluded if they had an open fracture, a tongue-type fracture, a non-displaced fracture (Sanders type 1), or had been followed up for less than one year. The study received approval from the Institutional Ethics Committee (IEC 1538/2021), and all relevant regulations and guidelines were strictly followed (Figure 1).

### 2.2. Treatment Methods

The respective treatment method was decided by the foot surgeon on duty.

Patients treated conservatively were immobilized with a lower leg cast and forearm crutches for 6–8 weeks. Operative treatment consisted of one of these two methods: (1) percutaneous reduction and fixation with a two-point distractor (ITS Implants, Autal, Laßnitzhöhe, Austria) according to Fröhlich et al. [11], using cannulated cancellous screws and K-wires, or (2) open reduction and internal fixation (ORIF) using plates, cannulated cancellous screws, and K-wires. All surgeries were performed by senior surgeons with extensive expertise in foot surgery. The treatment choice among the two surgical techniques was based on the preferences and experience of the surgeons involved in the operations and on the basis of the soft tissue condition of the single cases.

**Figure 2:**
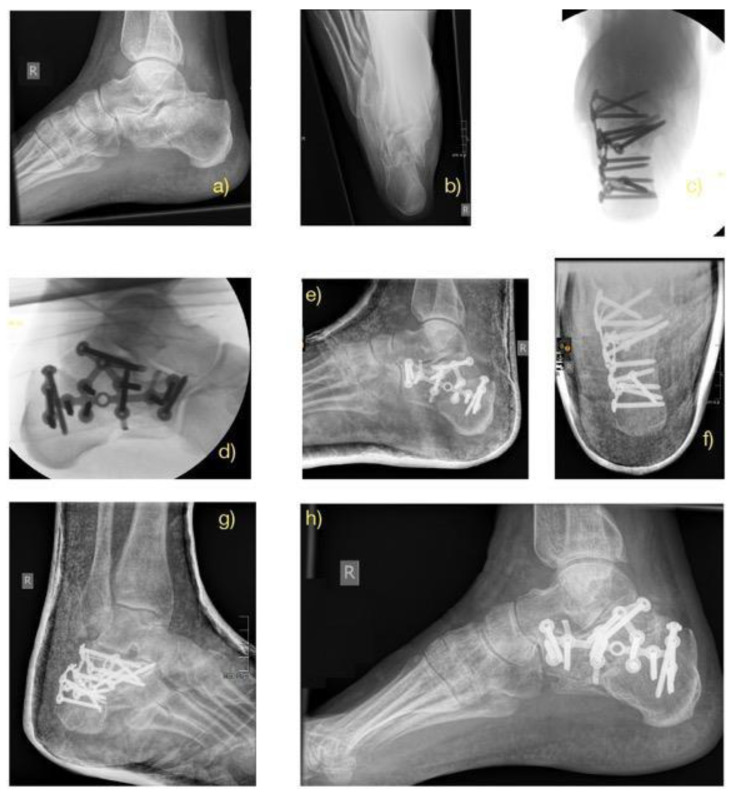
ORIF Case: 62-year-old male patient after a fall on the right calcaneus. Preoperative X-ray lateral (**a**) and axial (**b**): intra-articular calcaneal fracture with pathological flattening of the Böhler’s angle and valgization of the right hindfoot. Intraoperative pictures (**c**,**d**). Follow-up after 14 days. (**e**–**g**) and Follow-up after 12 months (**h**).

**Figure 3:**
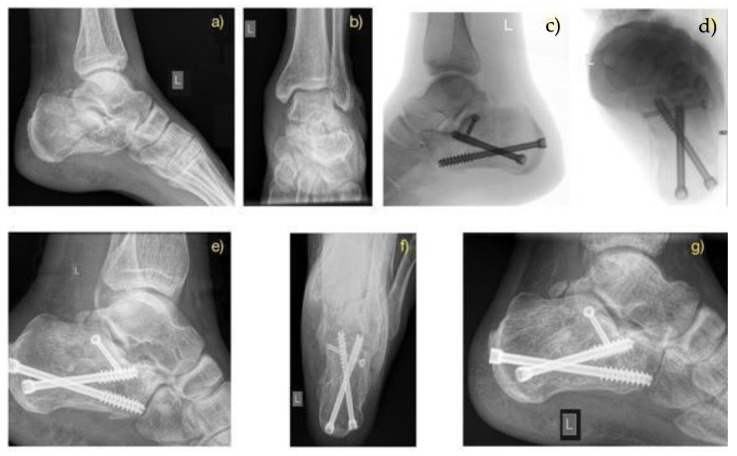
Fracture Case treated with minimal invasive technique according to Fröhlich at al. Preoperative X-ray: lateral (**a**) and axial (**b**), Intraoperative pictures (**c**,**d**), 14-day postoperative X-ray of left hindfoot (**e**,**f**). Follow-up after 12 months (**g**).

**Figure 4:**
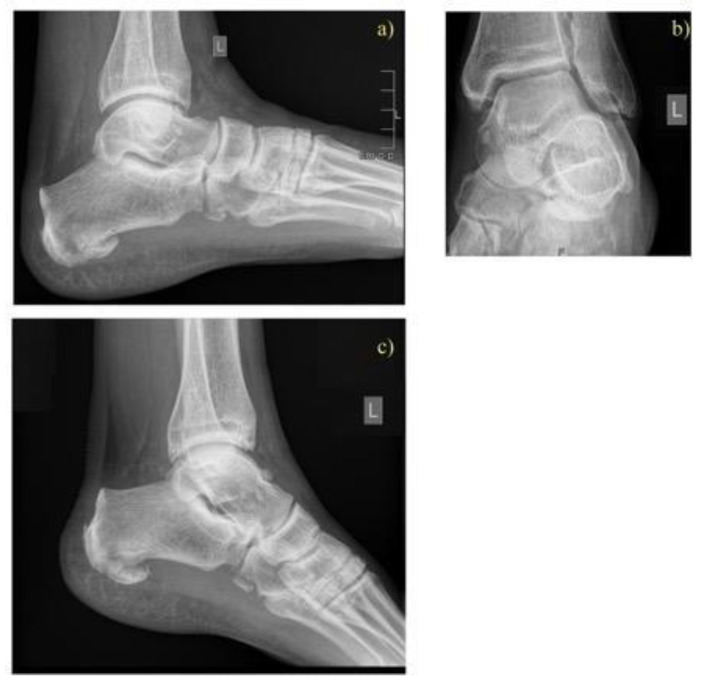
Conservative case: 43-year-old patient with bilateral calcaneal fracture after a fall. The left foot showed a slight dislocation of a fracture fragment (Sander type II) on X-ray (**a**,**b**). Follow-up after 10 months (**c**).

Patients in both surgical groups started exercise regimens for the foot and ankle joints based on their pain level one day after surgery. Initial partial weight-bearing was recommended for eight weeks postoperatively, followed by full weight-bearing after 12 weeks, provided radiographic union was achieved (Figure 2, Figure 3 and Figure 4).

### 2.3. Evaluation

Follow-up checks were performed at regular intervals and included clinical examination, wound checks, dressing changes, radiological assessments based on X-ray images with follow-up Böhler’s (measured line between the highest point of the anterior process and posterior articular facet with another line joining the highest point of the posterior articular facet with the highest point of the calcaneal tuberosity) and Gissane’s (measured by the intersection of a line from the highest point of the posterior articular facet to the highest point of the posterior tuberosity with another line from the former to the highest point of the anterior articular facet) angles,. Documentation and position control of the osteosynthesis material. Mean follow up time for all patients was 1.5 years, minimum 1.1 years and maximum 8.0 years.

The clinical outcomes included the visual analogue scale (VAS), the (AOFAS) Ankle-Hindfoot Score, Parker Mobility Score (PMS), and the Maryland Foot Score (MFS) were used to evaluate the treatment results. To avoid interobserver bias, the values were checked by two authors.

Evaluation measurements were taken from 1 year follow up and used for further analysis.

The statistical analyses were carried out using the program R. version 4.0.3. Data analysis was done using the Student’s unpaired *t*-test and Anova test. The Wilcoxon paired rank test and Mann-Whitney U was used for paired data, such as longitudinal comparison within each group for Böhler’s angle and Gissane’s angle. A Cochran-Mantel-Haenszel test was also calculated to investigate the difference in treatment by center. The correlation between treatment method and the onset of complications was tested with multivariate logistic regression; the odds ratio and its 95% confidence interval were calculated. A *p*-value ≤ 0.05 was considered significant.

Interobserver results were analyzed using Kendall’s W (Kendall coefficient of concordance W). Range of values of a Kendall’s W is from 0–1. A 0 value indicates no concordance among a set of raters while a score of 1 indicates perfect concordance.

## 3. Results

A total of 236 patients with 256 fractures were included, comprising 175 men and 61 women, with a mean age of 45.7 (SD 15.6) years. The majority of fractures were caused by a fall from height (*n* = 190) while 18 resulted from road traffic accidents. According to the Sanders classification, there were 33 type II, 79 type III, and 144 type IV fractures (Table 1).

The majority of patients who received surgical treatment had Sanders type IV fractures. These were treated almost equally with ORIF and MIT, whereas Sanders type II and III fractures were predominantly treated using minimally invasive techniques. Surgical time and Length of stay was significantly shorter within the MIT group (Table 1).

Both surgical cohorts resulted in a satisfactory reduction, as shown by acceptable ranges of Böhler’s angle at all time points and significant for Sanders 3 and 4 fractures, when compared with the conservative cohort (Table 2 and Table 3).

Preoperatively, the average Bohler’s angle for Sanders fracture 3 and 4 was 111.4 ± 11.5 and 107.9 ± 19.8 for the ORIF group and 111.6 ± 11.4 and 106.1 ± 15.7 degrees for MIT. In the immediate postoperative period, the average Bohler’s angle was 113.7 ± 9.1 and 118.6 ± 8.9 degrees and 118.6 ± 6.5 and 115.9 ± 11.3 for the ORIF and MIT groups, respectively. At the final follow-up, the average measured Böhler’s angle was 111.9 ± 18.1 and 116.0 ± 8.1 degrees for the ORIF group and 116.8 ± 4.9 and 111.5 ± 11.9 for the MIT group. Examples of pre- and postoperative Bohler’s angles for MIT and ORIF and without surgery in the CT group can be seen in Figure 1, Figure 2, and Figure 3, respectively.

### 3.1. Complications

The complications in our study included wound healing disorders, infections, revisions, gait disorders, and arthrosis.

Most wound healing disorders and infections were recorded in the ORIF group, and the highest proportion of arthrosis was found in the conservative group. Stiffness and gait abnormalities were the most common complications in all 3 treatment groups (Table 4).

Multivariate logistic regression showed a significant correlation between the treatment methods and occurrence of complications (Table 5).

### 3.2. Evaluation of the Scores

At the one-year follow-up, surgically treated fracture cases showed significantly better functional outcomes with MFS and VAS. AOFAS and PMS showed moderate to high significance. Spearman analysis showed a negative correlation between VAS and AOFAS (Table 6).

The intra-observer concordance, W, showed 0.7, which is a good agreement between both observers.

## 4. Discussion

The calcaneus is an essential weight-bearing bone and is often associated with higher morbidity in cases of highly comminuted fractures. However, there is still no clear consensus within the clinical community on the best treatment approach. Although ORIF provides better anatomical reduction, its major drawbacks persist, with wound complications and infections reported in up to 30% of cases. To mitigate these issues, various minimally invasive techniques have been developed. As a result, percutaneous reduction and fixation with cannulated screws have gained popularity for treating calcaneal fractures. These minimally invasive approaches offer several advantages, including reduced morbidity, faster recovery, a shorter rehabilitation period, and less tissue trauma.

### 4.1. Quality of Anatomical Reductions

In terms of anatomical reduction, the radiological Böhler’s and Gissane’s angles are often used as measurements of quality [5]. Studies have shown better long-term results after restoring the physiological Böhler’s angle [12,13]. Some authors also attribute a prognostic factor to Böhler’s and Gissane’s angles, which can be used to estimate the postoperative outcome at admission [14]. The total patient cohort showed significant differences between the preoperative and postoperative/follow-up measurement results. The observed postoperative differences and increases in Böhler’s and Gissane’s angles are comparable to the results described in the literature [10]. The operated patient cohort (ORIF and MIT) showed better radiological results than the conservative cohort.

### 4.2. Complications Within the Three Cohorts

Regarding complications, surgical procedures (MIT and ORIF) compared to conservative treatment are a frequently discussed problem in the literature [15,16]. When comparing the surgical groups with each other, serious complications such as wound healing disorders, soft tissue infections, revisions and arthrodesis are more frequently observed, with open reduction procedures than with minimally invasive procedures [17,18]. In our study, wound healing disorders were observed more frequently in the ORIF group than in the MIT group in both centers. This observation was also made by other authors [19,20]. The infection rate in all three treatment groups was consistent with the results found in other studies [10,21]. Smoking, diabetes, Sanders fracture type, but also the duration of surgery are discussed as risk factors for wound healing disorders and infections. In our study, surgery time, time until surgery and fracture type were significantly associated with the onset of complications. Since type IV Sanders fractures with longer operation times were observed more frequently in the ORIF group than in the minimally invasive procedures, this could partly explain the higher complication rate in the ORIF group. Revisions and osteoarthritis were observed in our study with approximately the same frequency as in the literature [20]. Overall, our study confirms the significant reduction in wound complications with the minimally invasive method compared to ORIF.

### 4.3. Functional Outcome

Clinical scores for calcaneal fractures are a topic of debate in the literature, as the authors often observe different results. A prospective study evaluating the AOFAS and VAS scores in favor of open reduction found advantages in terms of clinical-functional outcomes compared to the minimally invasive method [22]. The results of our study showed comparable AOFAS scores without statistically significant differences between the treatment methods. However, median values of the AOFAS score in our study showed a tendency towards slightly higher values for the surgical treatment groups. This was similar to the results reported by De Boer et al., who found better AOFAS scores in the ORIF and MIT groups, but overall these did not differ significantly from the conservative treatment group [20]. Other studies comparing the ORIF and MIT methods also found no significant differences between the AOFAS scores after ORIF and minimally invasive methods [3]. A meta-analysis by Fan et al. supports our results by finding no difference in functional outcomes between the different surgical methods as measured by the AOFAS score [17]. These results, in accordance with our study, suggest that both minimally invasive and classic open reduction and internal fixation can achieve comparable results in terms of functional recovery after calcaneus fractures. The minimally invasive method is being used more frequently, leading to improvements in surgical procedures and better long-term results for patients. With regard to the VAS score, studies have shown controversial results within surgical treatments. According to Biz et al., better VAS scores were achieved when patients received good education about their injury and were informed about the treatment options [19].

Other authors who compared surgical and non-surgical treatment found equivalent results between the two groups, similar to our observations. Agren et al. found no differences between operated and non-operated patients in the 1-year follow-up. However, during further follow-up after 8–12 years, the operated patients showed better AOFAS values, although without statistically significant differences [14]. Similar results have been shown for MFS in operated patients [23]. Accordingly, the results in the literature show a tendency towards better long-term outcomes after surgical reconstruction, and our results confirm this observation. For the Parker Mobility Score, no studies were found that compared this score for calcaneus fractures. Our study showed equivalent results with all three treatment methods.

Khurana et al. noted a correlation between the AOFAS score and the postoperative Böhler’s angle, with higher angles associated with better scores [22]. In contrast, Biz et al. found no correlation between the postoperative Böhler’s angle and the AOFAS and VAS scores, concluding that Böhler’s angle is an inappropriate radiological measurement. Our results confirm the findings reported by Biz et al. [19].

Furthermore, we observed better anatomical reductions after surgery, and in terms of clinical scores, patients achieved slightly better clinical rehabilitation when surgically treated. Additionally, MIT showed significantly fewer complications compared to ORIF.

For severely comminuted Sanders type IV calcaneal fractures, ORIF tends to provide more stable support for the articular surface, minimizing the need for extensive bone grafting in cases of articular collapse despite the higher risk of postoperative complications. Nevertheless, radiological differences between both surgical groups in our study were not significant, proving MIT to be a good alternative, especially for Sanders type IV fractures, which tend to have more complications.

A major limitation of our study is the retrospective nature and the associated disadvantages. The distribution of patient numbers across the treatment groups was not evenly distributed, and furthermore, the variation in surgical treatment concerning Sanders classification and follow-up time was inconsistent, making it difficult to compare the patient groups with each other and with other studies. Our study’s findings should be validated through further multicenter studies with long-term outcome evaluations.

Furthermore, both centers did not obtain postoperative CT scans on all patients due to costs and the need for the utilization of resources, and we did not evaluate any peripheral nerve damage, which is common in foot and ankle surgery

## 5. Conclusions

Surgically treated patient groups benefit from better anatomical reductions compared to conservative patients despite higher wound complication rates. Clinical and functional results were significantly better than compared to conservative treatment at one year. Overall, surgical interventions are preferable to conservative treatment for Sanders types III-IV fractures. Minimally invasive treatments proved to be equivalent to open reduction and internal fixation in Sanders types III-IV fractures in terms of their anatomical reconstruction and functional results and showed less complications, shorter surgery times and lengths of stay.

## Figures and Tables

**Table 1:** Overview of the demographic and clinical data.

	ORIF	MIT	CT	*p*
Number of patients	60	70	106	0.02
Age (mean ± standard deviation)	45.1 ± 13.1	48.8 ± 13.1	44.8 ± 18.5	0.04
Gender				
Male	53	157	67	0.2
Female	14	43	28	
Fracture side				0.4
Unilateral	50	40	70	
Bilateral	5	15	28	
Cause of accident				0.008
Fall	47	51	86	
Road accident	12	15	10	
Other causes	1	4	10	
Sanders Classification				0.3
2	1	10	19	
3	22	21	41	
4	37	39	66	
Surgery time (minutes)				
(mean ± standard deviation)	119.1 ± 39.3	51.3 ± 29.2	n/a	*p* < 0.001
Length of stay (days)				
(mean ± standard deviation)	15.7 ± 12.8	10.5 ± 14.1	13.5 ± 19.5	*p* < 0.001

**Table 2:** Overview of Böhler’s angle measurement for ORIF, MIT, and CT cohort.

Böhler’s Angle	ORIF	MIT	CT	*p*
preoperative				
Sanders 2	26.6 ± 8.3	25.4 ± 5.6	30.8 ± 6.9	0.1
Sanders 3	19.3 ± 7.1	23.3 ± 4.9	25.4 ± 8.7	0.1
Sanders 4	20.8 ± 7.5	20.6 ± 6.0	23.9 ± 7.0	0.4
postoperative				
Sanders 2	28.6 ± 7.1	29.4 ± 5.1	n/a	0.4
Sanders 3	27.7 ± 4.9	29.8 ± 5.7	n/a	*p* < 0.001
Sanders 4	27.9 ± 5.0	28.6 ± 5.9	n/a	*p* < 0.001
follow-up				
Sanders 2	27.5 ± 4.5	28.3 ± 4.4	26.4 ± 7.8	0.5
Sanders 3	26.5 ± 4.8	28.8 ± 5.9	20.9 ± 8.8	*p* < 0.001
Sanders 4	25.6 ± 4.9	26.2 ± 5.8	18.5 ± 10.9	*p* < 0.001

**Table 3:** Overview of Gissane’s angle measurement for ORIF, MIT, and CT cohort.

Gissane’s Angles	ORIF	MIT	CT	*p*
preoperative				
Sanders 2	111.7 ± 25.9	110.4 ± 12.3	100.4 ± 20.5	0.9
Sanders 3	111.4 ± 11.5	111.6 ± 11.4	122.6 ± 10.5	0.8
Sanders 4	107.9 ± 19.8	106.1 ± 15.7	109.4 ± 16.9	0.9
postoperative				
Sanders 2	120.7 ±15.4	122.4 ± 9.5	n/a	0.5
Sanders 3	113.7 ± 9.1	118.6 ± 6.5	n/a	*p* < 0.001
Sanders 4	118.6 ± 8.9	115.9 ± 11.3	n/a	*p* < 0.001
follow-up				
Sanders 2	119.9 ± 14.3	120.7 ± 7.8	98.5 ± 17.8	0.8
Sanders 3	111.9 ± 18.1	116.8 ± 4.9	110.2 ± 11.2	*p* < 0.001
Sanders 4	116.0 ± 8.1	111.5 ± 11.9	104.8 ± 10.5	*p* < 0.001

**Table 4:** List of complications.

Complications	ORIF	%	MIT	%	CT	%	*p*
	*n* = 60		*n* = 70		*n* = 126		
Wound healing disorders	10	16.7	5	7.1	1	0.8	0.7
Deep Infection	7	11.7	2	2.9	1	0.8	*p* < 0.001
Revision	3	5.0	1	1.4	0		1
Stiffness	15	25.0	18	25.7	22	17.5	0.07
Gait abnormality	7	11.7	5	7.1	34	27.0	0.07
Osteoarthritis Grade 3	5	8.3	3	4.3	37	29.4	0.09

**Table 5:** Multivariate analysis of complication.

	OR	95% CI		*p*
Days until surgery	1.033	0.969	1.101	0.32
Surgery time	0.86	0.472	1.579	0.63
Sanders fracture	1.391	1.259	1.536	*p* < 0.001
Surgical Therapy	0.607	0.457	0.807	*p* < 0.001
Conservative Therapy	0.513	0.535	0.787	*p* < 0.001

**Table 6:** Clinical scores at the one-year follow-up.

Score (Mean ± SD)	ORIF	MIT	CT	*p*
MFS	72.2 ± 10.4	76.3 ± 9.8	55.4 ± 6.5	*p* < 0.001
AOFAS	79.0 ± 10.6	81.7 ± 9.5	71.8 ± 13.2	*p* = 0.04
VAS	3.6 ± 0.6	3.3 ± 0.9	4.3 ± 1.7	*p* < 0.001
PMS	8.7 ± 0.5	8.4 ± 1.0	7.4 ± 1.7	*p* = 0.004

## Data Availability

The datasets used and/or analyzed during the current study are available from the corresponding author on reasonable request.
